# In Silico and In Vitro Elucidation of the Inhibitory Mechanism of Chlorogenic Acid Against *Helicobacter pylori* Urease

**DOI:** 10.1002/fsn3.72065

**Published:** 2026-06-28

**Authors:** Luzhe Wu, Shengyao Hou, Hongguang Cao, Chenyu Wang, Ning Xu, Chen Yu, Yanni Li

**Affiliations:** ^1^ School of Pharmacy Binzhou Medical University Yantai Shandong China; ^2^ Shandong Qidu Pharmaceutical Co. Ltd Zibo Shandong China; ^3^ The First School of Clinical Medicine Binzhou Medical University Yantai Shandong China

**Keywords:** chlorogenic acid, *Helicobacter pylori* urease, inhibition, molecular docking

## Abstract

Chlorogenic acid (CGA), a phenolic compound widely distributed in plants, has exhibited inhibitory activity against *Helicobacter pylori* ( *H. pylori* ) by effectively suppressing urease, a key virulence factor of the pathogen. This study aimed to investigate the binding affinity between CGA and urease and explore the underlying molecular mechanism. Inhibition kinetics were characterized using Lineweaver–Burk plots, fluorescence quenching, isothermal titration calorimetry (ITC), and surface plasmon resonance (SPR). The binding mode was elucidated using molecular docking and molecular dynamics simulations. The results demonstrated that CGA exhibited a mixed‐type inhibition against *H. pylori* urease (HPU). CGA quenched the intrinsic fluorescence of HPU via a static quenching mechanism, with the quenching constant (*K*_q_) of 0.41 ± 0.04 × 10^13^ M^−1^ s^−1^. The thermodynamic parameters indicated that the interaction between CGA and HPU was exothermic and enthalpy‐driven (Δ*G* < 0, Δ*H* < 0). SPR data revealed that CGA had a dissociation constant (*K*_D_) of 1.63 ± 0.19 × 10^−5^ M. CGA bound to HPU near the active site and flap region, forming a stable complex. These findings contribute to the understanding of phenolics in anti‐ *H. pylori* therapy and highlight CGA as a promising lead compound for the development of innovative urease‐targeted applications.

## Graphical abstract

Chlorogenic acid (CGA) inhibits *Helicobacter pylori* urease (HPU) through a mixed‐type mechanism. The CGA–HPU interaction is thermodynamically spontaneous and enthalpy‐driven (Δ*G* < 0, Δ*H* < 0), proceeding via a static quenching mode. Molecular dynamics simulations and structural studies reveal that CGA binds near the HPU active site and mobile flap region, engaging key residues Ala365, Met366, and Asp223. These findings provide novel mechanistic insights into CGA–HPU interactions and support the rational design and optimization of CGA and related hydroxycinnamic acids as potent urease inhibitors.

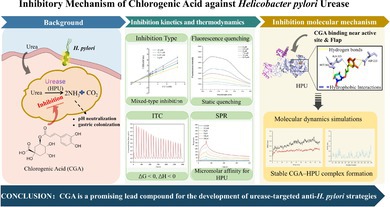

## 1 Introduction

*Helicobacter pylori* ( *H. pylori* ) is a highly prevalent human pathogen that mainly colonizes the gastric epithelium. Currently, *H. pylori* infects approximately 50% of the global population (Gupta et al. 2023, 2025; Kunkalienkar et al. 2025; Saha et al. 2025). Persistent infection leads to chronic active gastritis, peptic ulcer disease in 1%–10% of cases, and gastric cancer in about 0.1%–3% of cases (Gonzalez‐Hormazabal et al. 2021; Sugano 2016). Recently, eradication success has declined due to rising antimicrobial resistance, poor patient compliance, and adverse side effects (Chey et al. 2017; Howden and Graham 2021; Jung et al. 2021; Liou et al. 2016). Consequently, targeting *H. pylori* virulence factors offers a selective therapeutic strategy that minimizes disruption to the gut microbiota and reduces the risk of resistance.

Urease is a key virulence factor that enables *H. pylori* to survive and colonize the hostile acidic gastric environment by catalyzing urea hydrolysis to neutralize local pH (Murata‐Kamiya and Hatakeyama 2022; Gonciarz et al. 2019). The byproduct ammonia compromises the gastric epithelial barrier by disrupting tight junctions and activating NF‐κB signaling, which subsequently promotes IL‐8‐mediated neutrophil recruitment and chronic inflammation (Wroblewski et al. 2009; Beswick et al. 2006). Structural insights from X‐ray crystallography highlight the nickel active site and the mobile flap as essential drug targets (Mazzei et al. 2020). Potent inhibitors function by blocking the active site, acting as substrate mimics, or interfering with the flap's open–closed transitions (Yang et al. 2018; Lu et al. 2022).

Chlorogenic acid (CGA), a dietary polyphenol commonly found in coffee and fruits, has been identified as a potent urease inhibitor (Ertas et al. 2021; Xiao et al. 2012). Kataria and Khatkar reported that CGA exhibited an inhibitory potency (IC_50_ = 22.68 ± 0.006 μM) (Kataria and Khatkar 2019). Furthermore, the urease inhibitory activity of numerous plant extracts was closely linked to their phenolic content, with CGA frequently identified as a primary bioactive constituent (Ahmad et al. 2021; Lekmine et al. 2025; Azhikhanova et al. 2024). Molecular docking suggested that CGA may exert its effects by interacting with the flap (Thr374) of *H. pylori* urease (HPU, PDB 8HC1) (Haq et al. 2024). Previous work in our laboratory demonstrated the in vivo anti‐urease effects of dandelion extract and further elucidated the inhibitory mechanism of CGA on jack bean urease (Dong et al. 2024).

Although CGA has been identified as a urease inhibitor, its binding mechanism remains insufficiently characterized. In this study, we combine enzymatic, biophysical, and computational approaches to characterize the CGA–HPU interaction, linking binding affinity with thermodynamic and kinetic properties. These results provide mechanistic insights that may facilitate the rational design of improved urease inhibitors.

## 2 Materials and Methods

### 2.1 Chemicals and Reagents

CGA (≥ 98%, catalog no. 240613) was purchased from Chengdu Purechem‐Standard Co. Ltd. (Chengdu, China). HPU was extracted from *H. pylori* strain 26,695, which was purified by DEAE ion‐exchange chromatography using a HiTrap DEAE FF column. The column was equilibrated with 20 mM phosphate buffer (PB, pH 7.0) and eluted with a NaCl gradient (100–300 mM). Purity was verified by SDS–PAGE (Figure S1) (Liu et al. 2020). Purified HPU was stored at −80°C in 20 mM PB (pH 7.0) until use. All other reagents were analytical grade and commercially available.

### 2.2 Determination of Urease Inhibition Activity

A urease inhibition assay was performed using the Berthelot method (Sevgi et al. 2017). One unit of urease activity was defined as the amount of enzyme that releases 1 μmol of ammonia per minute at 37°C. Ammonia concentration was quantified using a standard curve. CGA was dissolved in phosphate buffer (0.1 M, pH 6.8) and diluted to final concentrations of 3.13, 6.25, 12.5, 25, and 50 μM. The reaction mixture contained 500 μL urea (30 mM), 200 μL HPU solution, and 200 μL CGA (3.13–50 μM). The mixture was incubated for 30 min. Then, 500 μL of Berthelot reagent A and 600 μL of reagent B were added. The mixture was incubated in the dark for 10 min, and absorbance was measured at 625 nm after subtracting the buffer control. The final volume of the reaction mixture was 2 mL. Urease activity in the absence of inhibitor was defined as 100%. All measurements were performed in triplicate, and acetohydroxamic acid (AHA) was used as the positive control. The half‐maximal inhibitory concentration (IC_50_) was determined by plotting the percentage of HPU activity against inhibitor concentration.

### 2.3 Inhibition Kinetics Assay

The inhibition kinetics of CGA against HPU were determined as previously described (Lu et al. 2022). A 900 μL reaction mixture containing 200 μL HPU, 200 μL CGA, and 500 μL urea (0.313–10 mM) was prepared and incubated at 37°C for 30 min. The amount of ammonia generated was then quantified using the Berthelot method, as described in Section 2.2, to determine urease activity. Lineweaver–Burk plots of 1/*v* versus 1/[*S*] were constructed to determine the inhibition type and estimate the Michaelis constant (*K*_m_) and maximum velocity (*V*_max_).

### 2.4 Fluorescence Quenching Assay

The interaction between CGA and HPU was investigated using fluorescence quenching spectroscopy (Kyrychenko and Ladokhin 2024). Briefly, HPU was incubated alone or with CGA at different concentrations (0–12.5 μM) for 10 min at 298 K. To minimize potential inner filter effects, CGA concentrations were kept low (0–12.5 μM), and all samples were prepared with the same solvent composition and measured under identical instrumental settings. Emission spectra were recorded in the range of 300–360 nm with an excitation wavelength of 280 nm, with excitation and emission bandwidths of 5 nm. The quenching mechanism was assessed using the Stern–Volmer equation:

(1) $$F_{0}/F=1+K_{\mathrm{sv}}\left[Q\right]=1+K_{q}\times \tau_{0}\;\left[Q\right]$$

*F*_0_ and *F* are the fluorescence intensities of HPU in the absence and presence of CGA, respectively; *K*_sv_ is the Stern–Volmer quenching constant; *K*_q_ is the bimolecular quenching rate constant; *τ*_0_ is the average fluorescence lifetime of the protein in the absence of quencher (*τ*_0_ = 10^−8^ s) (Lakowicz 2006); and [*Q*] is the molar concentration of CGA.

To obtain the binding constant (*K*) and the number of binding sites (*n*), the following equation was employed:

(2) $$\log\;\left(F_{0}-F\right)/F=\log\;K+n\;\log\;\left[Q\right]$$

### 2.5 Isothermal Titration Calorimetry (ITC)

Thermodynamic parameters of CGA‐HPU binding were measured by ITC using Nano ITC (TA Instruments, New Castle, DE, USA) (Gal et al. 2016). Prior to measurement, HPU was dissolved in 20 mM phosphate buffer (pH 7.0) to a final concentration of 20 μM, while CGA was prepared in the same buffer at 500 μM. All solutions were degassed prior to use. The sample cell was loaded with HPU, followed by titration with 2.5 μL injections of CGA solution at 298 K. The interval between successive injections was maintained at 150 s, with a stirring speed of 300 rpm. The data were analyzed using a single‐site binding model to determine the thermodynamic parameters, including the dissociation constant (*K*_d_), the association constant (*K*_a_), enthalpy change (Δ*H*), entropy change (Δ*S*), and binding stoichiometry (N). The Gibbs free energy (Δ*G*) was calculated using the equation Δ*G* = Δ*H* − *T*Δ*S*.

### 2.6 Surface Plasmon Resonance Measurement (SPR)

SPR experiments were performed using a Biacore T200 instrument (Abuasaker et al. 2023). HPU (50 μg/mL in 10 mM sodium acetate buffer, pH 4.0) was immobilized on a CM5 sensor chip via standard amine coupling to a level of 14,000 RU (Cytiva, Marlborough, MA, USA). CGA at six concentrations (6.25–200 μM) was injected over the chip surface in running buffer (PBS containing 0.05% Tween 20) at a flow rate of 10 μL/min. The contact and dissociation times were 120 and 600 s, respectively, at 298 K. The surface was regenerated with 10 mM glycine–HCl (pH 3.0) for 30 s between injections. Sensorgrams were processed using BIAevaluation software (GE Healthcare, Chicago, IL, USA) with double referencing (reference channel subtraction and blank buffer correction). Buffer matching was applied during sample preparation, and buffer‐only injections were used as controls. The data were fitted to a 1:1 Langmuir binding model to determine the kinetic rate constants (*K*_on_ and *K*_off_). The equilibrium dissociation constant (*K*_D_ = *K*_off_/*K*_on_) and the corresponding equilibrium association constant (*K*_A_ = 1/*K*_D_) were derived from the fitted kinetic rate constants.

### 2.7 Protein‐Inhibitor Preparation and Molecular Docking

Molecular docking was performed using AutoDock 4.2.6 (Qi et al. 2022). The 3D structure of CGA was retrieved from PubChem, and the HPU structure (PDB ID: 1E9Y) was obtained from the Protein Data Bank. The file of 1E9Y contains two protein chains, chain A and chain B, corresponding to the UreA and UreB subunits, respectively; therefore, the αβ heterodimeric catalytic unit was used in this study. HPU was prepared by removing water molecules, adding polar hydrogens, and assigning Kollman charges. The grid box was centered on the binuclear nickel active site with dimensions of 60 × 60 × 60 points and a spacing of 0.375 Å. A total of 50 docking runs were performed using the Lamarckian genetic algorithm (LGA). The resulting docking poses were analyzed and visualized using PLIP and PyMOL for binding interaction analysis.

### 2.8 Molecular Dynamics Simulations

Molecular dynamics simulations were carried out to analyze the detailed interaction mechanism between the inhibitor and urease at the molecular level (Amin et al. 2022). The lowest‐energy docked conformation of CGA was selected and processed using the Antechamber Python Parser interface (ACPYPE) to generate the required topology files. Simulations were carried out using GROMACS 2020.5 with the AMBER99 force field and the TIP3P water model (Abraham et al. 2015). After the system was neutralized with Cl^−^ ions and energy‐minimized using the steepest descent method, a 1 ns NVT equilibration at 300 K with position restraints was performed, followed by a 50 ns NPT simulation. The system temperature was maintained at 300 K using a V‐rescale thermostat, while long‐range electrostatic interactions were treated using the particle mesh Ewald (PME) method (Darden et al. 1993). The LINCS algorithm was used to constrain bond lengths with a time step of 2 fs, and default values were used for all other parameters. The stability of the CGA–HPU complex was evaluated by analyzing the root mean square deviation (RMSD), root mean square fluctuation (RMSF), hydrogen bonds, and radius of gyration (Rg) of the system.

### 2.9 Binding Free Energy (MM‐PBSA) of the CGA–HPU Complex

The binding free energy was calculated with the Molecular Mechanics/Poisson–Boltzmann Surface Area (MM‐PBSA) approach using GROMACS 2020.5. To characterize the energetic contributions to CGA–HPU binding, the individual energy components were decomposed as follows: van der Waals energy (VDWAALS), electrostatic energy (EEL), polar solvation energy (EGB), and non‐polar solvation energy (ESURF), along with the gas‐phase interaction energy (GGAS), total solvation free energy (GSOLV), and the total binding free energy.

### 2.10 Statistical Analysis

Statistical analyses were conducted utilizing IBM SPSS software (IBM Corp., Armonk, NY, USA), with all results presented as mean ± SD. Differences among groups were assessed through a one‐way ANOVA, followed by Dunnett's test. *p* < 0.05 was considered statistically significant.

## 3 Results

### 3.1 Enzyme Inhibitory Kinetic Analysis of CGA

**FIGURE 1:**
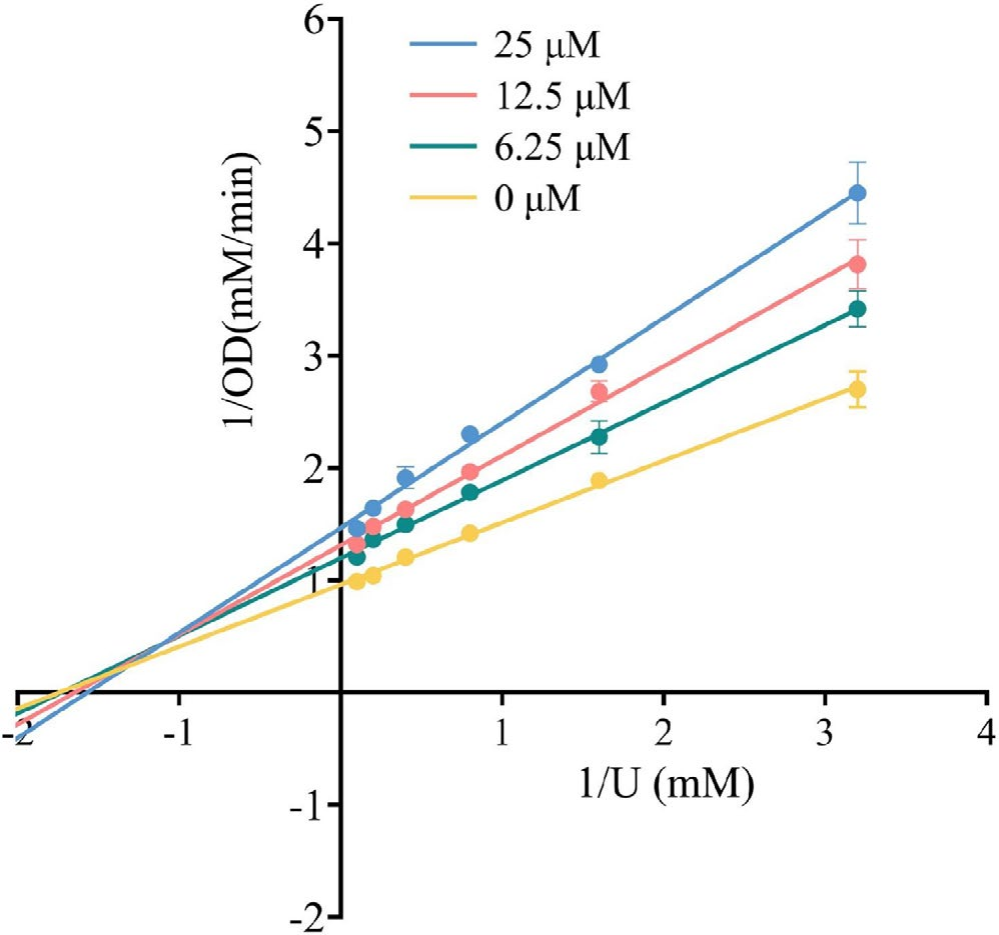
Lineweaver–Burk plot of HPU in the presence of CGA.

CGA exhibited an IC_50_ of 36.37 ± 3.34 μM (Figure S2), comparable to the positive control AHA (16.41 ± 1.31 μM), indicating moderate inhibitory activity. Enzymatic kinetics were assessed by nonlinear regression applied to the Michaelis–Menten equation in the absence or presence of different concentrations of CGA. The inhibition pattern was also determined by Lineweaver–Burk plot analysis. As shown in Figure 1 and Table S1, increasing concentrations of CGA led to a concurrent decrease in *V*_max_ and an increase in *K*_m_, suggesting a mixed‐type inhibition mechanism. This analysis yielded inhibition constants of *K*_i_ = 31.18 ± 5.62 and *K*_i_′ = 47.21 ± 9.94 μM. The calculated *α* value (*α* = *K*_i_′/*K*_i_) of 1.51 further corroborated the mixed inhibition profile.

### 3.2 Fluorescence Quenching Analysis of the CGA–HPU Interaction

**FIGURE 2:**
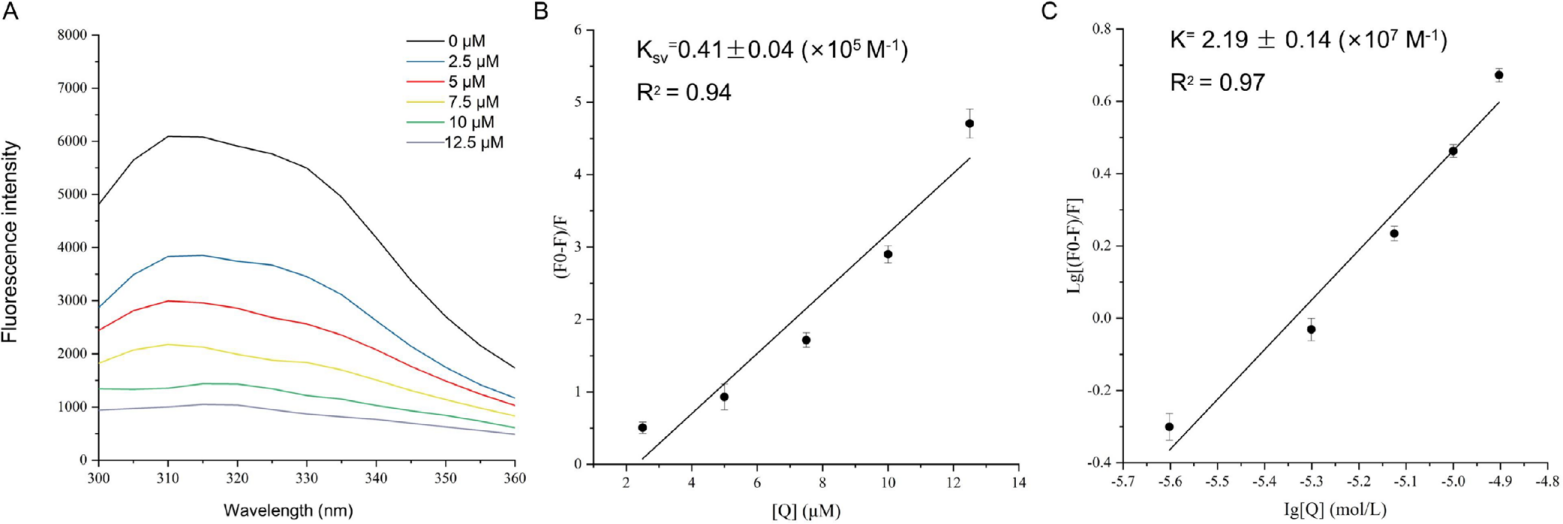
Fluorescence spectra of HPU in the presence of CGA. (A) Fluorescence emission spectra. (B) Stern–Volmer plot. (C) Double‐logarithmic plot.

HPU was titrated with different concentrations of CGA, and the fluorescence spectra were shown in Figure 2. The quenching data were analyzed using the Stern–Volmer equation. The Stern–Volmer quenching constant was *K*_SV_ = 0.41 × 10^5^ M^−1^ (*R* ^2^ = 0.94), and the bimolecular quenching rate constant was calculated as *K*_q_ = *K*sv/*τ*_0_ = 0.41 ± 0.04 × 10^13^ M^−1^ s^−1^, which far exceeded the diffusion‐controlled limit (2 × 10^10^ M^−1^ s^−1^), confirming a static quenching mechanism (Lakowicz 2006; Ma et al. 2017). As shown in Figure 2A, the maximum emission wavelength exhibited a slight red shift with increasing CGA concentration, suggesting subtle conformational changes in the microenvironment of the fluorophores upon CGA binding. In addition, the double‐logarithmic equation yielded an *n* value of 1.30 ± 0.14, consistent with a 1:1 binding stoichiometry between CGA and HPU.

### 3.3 Thermodynamic Properties of the CGA–HPU Interaction

**FIGURE 3:**
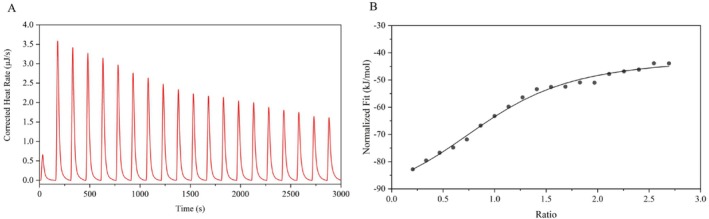
ITC analysis of CGA–HPU interaction. (A) Representative ITC titration curve. (B) Thermodynamic parameters of CGA–HPU binding.

As shown in Figure 3, the binding isotherm was fitted to obtain the dissociation constant (*K*_d_), thermodynamic parameters (Δ*H*, Δ*S*, and Δ*G*), and binding stoichiometry (*N*). The interaction exhibited a negative enthalpy change (Δ*H* = −53.36 ± 7.49 kJ mol^−1^) and a negative entropy change (Δ*S* = −80.59 ± 8.36 J mol^−1^ K^−1^), indicating that the binding was predominantly enthalpy‐driven with an unfavorable entropic contribution. At 298 K, Δ*G* was calculated from Δ*H* and Δ*S* via the relation Δ*G* = Δ*H*−*T*Δ*S*, yielding a value of Δ*G* = −29.33 ± 7.88 kJ mol^−1^. The binding stoichiometry *N* was 1.09 ± 0.17. The *K*_d_ (7.21 ± 0.86 × 10^−6^ M) and *K*_a_ (1.39 ± 0.17 × 10^5^ M^−1^) values indicated a binding affinity in the micromolar range.

### 3.4 Kinetic Properties of the CGA–HPU Interaction

**FIGURE 4:**
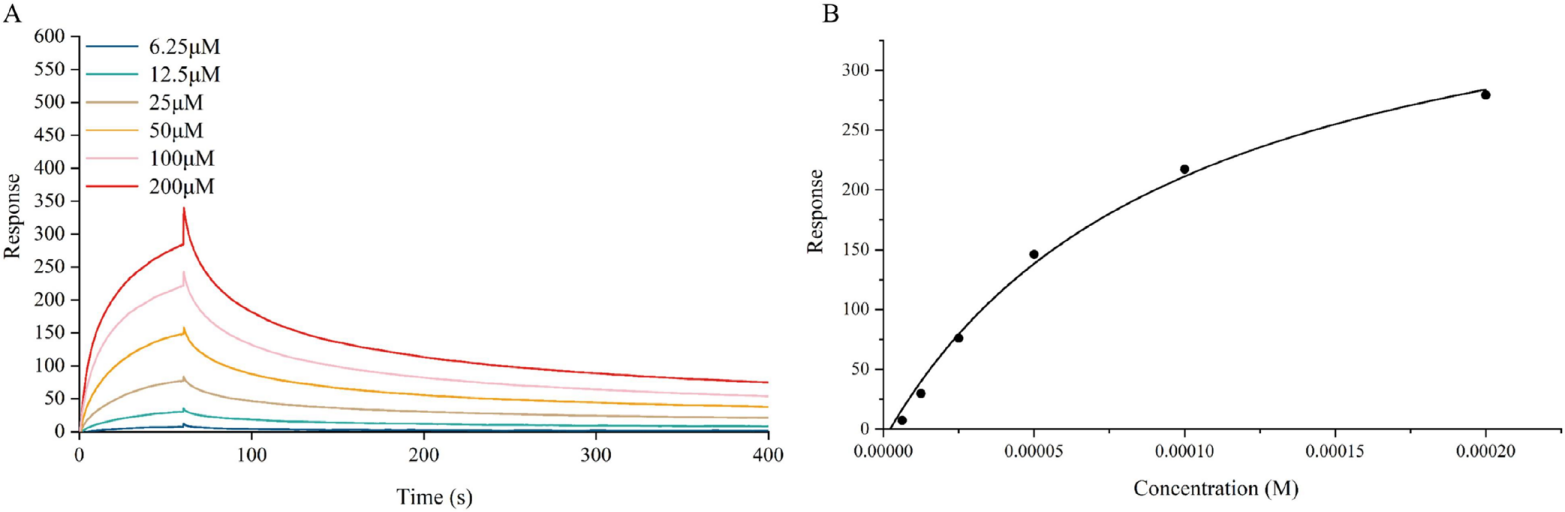
SPR analysis of CGA–HPU interaction. (A) Representative SPR sensorgram. (B) Equilibrium binding analysis of CGA–HPU interaction.

The interaction between CGA and HPU was analyzed using SPR. No obvious mass transport limitation was observed under the experimental flow rate. As shown in Figure 4, fitting of the sensorgrams to a 1:1 Langmuir binding model yielded a dissociation constant *K*_D_ of 1.63 ± 0.19 × 10^−5^ M (*K*_A_ of 6.18 ± 0.69 × 10^4^ M^−1^), indicating a moderate binding affinity between CGA and HPU. The association rate constant *K*_on_ and dissociation rate constant *K*_off_ were determined to be 1.68 ± 0.15 × 10^2^ M^−1^·s^−1^ and 2.73 ± 0.16 × 10^−3^ s^−1^.

### 3.5 Binding Mode Analysis of the CGA–HPU Complex

**FIGURE 5:**
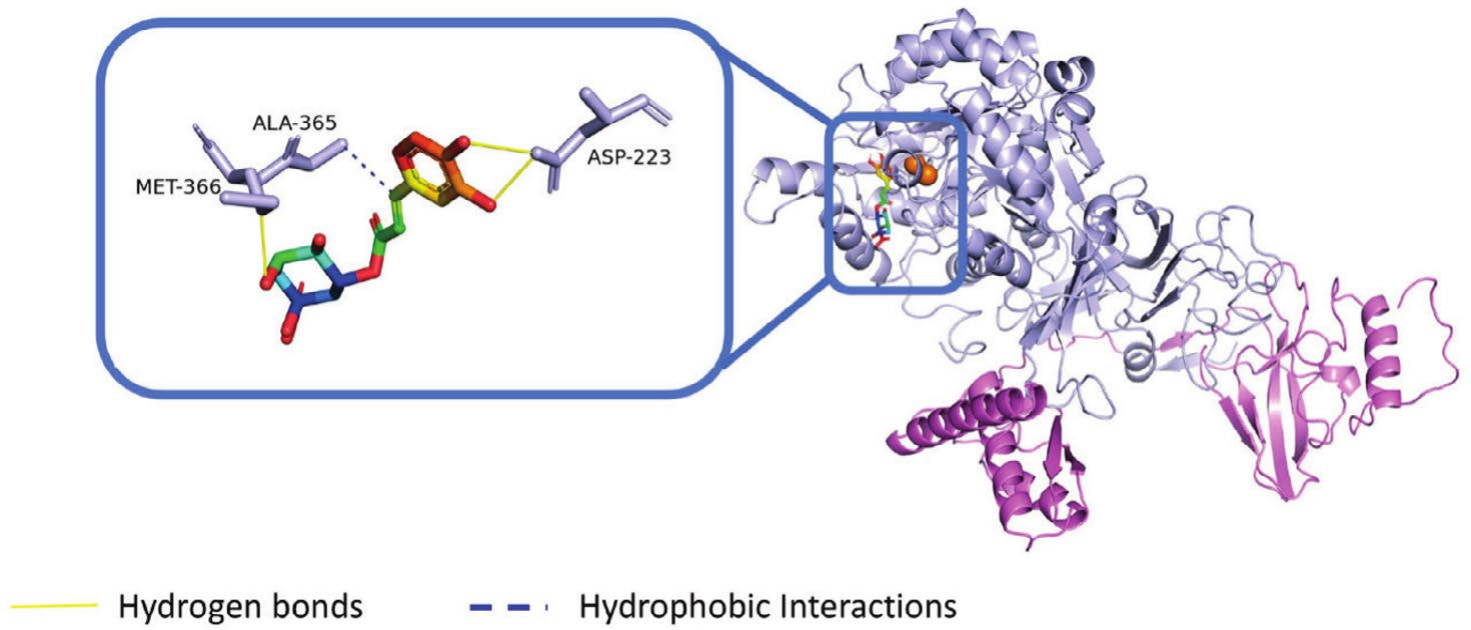
Molecular interactions between CGA and HPU.

The binding affinity of CGA to HPU was evaluated by molecular docking, yielding a binding energy of −5.63 kcal/mol. Figure 5 showed CGA positioned at the edge of the binding pocket, interacting with residues from both the active site and the mobile flap region. Hydrogen bonding plays a critical role in stabilizing protein‐ligand interactions. Three hydrogen bonds were identified: two were formed between the catechol hydroxyl groups of CGA and the carboxylate oxygen atoms of Asp223, and one was formed between the ester carbonyl oxygen of CGA and the backbone amide nitrogen of Met366. Hydrophobic interactions provide additional stabilization. CGA established a hydrophobic contact with Ala365, a residue located at the base of the mobile flap, through van der Waals forces between the cyclohexene ring of CGA and the methyl side chain of Ala365.

### 3.6 Dynamic Stability Analysis of the CGA–HPU Complex

**FIGURE 6:**
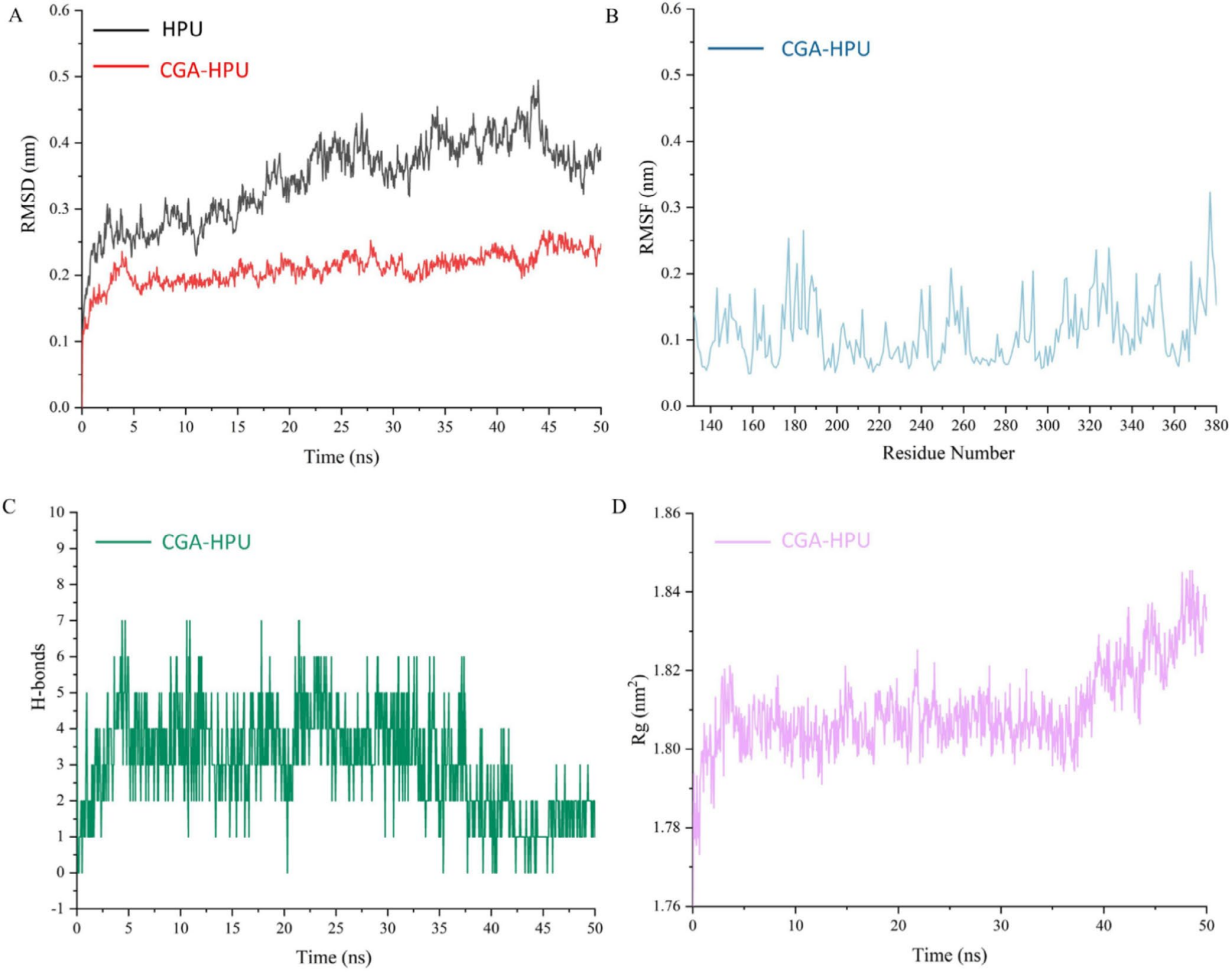
Molecular dynamics simulations of the CGA–HPU complex. (A) RMSD profiles of free HPU (black) and the CGA–HPU complex (red) over 50 ns; (B) RMSF of residues in the CGA–HPU complex; (C) Number of hydrogen bonds over 50 ns; (D) Rg of the CGA–HPU complex over 50 ns.

The structural stability and conformational changes of the CGA–HPU complex were evaluated using molecular dynamics simulations. As shown in Figure 6A, the RMSD of free HPU exhibited a gradual increase with significant fluctuations, reaching 0.45 nm by the end of 50 ns. In contrast, the CGA–HPU complex rapidly reached equilibrium, stabilizing at a significantly lower RMSD (average ~0.22 nm), which suggested that CGA binding enhances the structural rigidity of HPU.

RMSF analysis was used to quantify the local flexibility of the CGA–HPU complex (Figure 6B). Most residues maintained low fluctuations (< 0.25 nm), with the lowest values observed at key binding residues (Asp223 and Met366). The fluctuations within the flap region (residues 310–350) were significantly suppressed compared to other flexible segments. Hydrogen bond analysis (Figure 6C) revealed that the complex maintained 2–7 hydrogen bonds throughout the simulation, with transient peaks of up to 9 bonds, highlighting dynamic ligand–protein interactions. The compactness of the complex was assessed by analysis of the Rg (Figure 6D). The Rg slightly increased from 1.78 to 1.84 nm over 50 ns, indicating a subtle structural expansion or rearrangement. Furthermore, the binding free energy calculated via MM–PBSA was −16.63 ± 3.57 kcal/mol, confirming a thermodynamically favorable binding interaction (Das et al. 2023). The individual energy contributions were summarized in Table S2.

## 4 Discussion

*H. pylori* infection is a significant public health burden, with antimicrobial resistance increasingly compromising the efficacy of standard triple or quadruple eradication regimens (Wenker et al. 2023). Targeting urease represents a mechanistically distinct alternative that avoids direct antibiotic pressure. CGA is an abundant dietary polyphenol widely found in coffee, fruits, and vegetables, with high bioavailability and potent pharmacological activity. The present study aimed to provide a mechanistic rationale for its potential application as a food‐derived urease inhibitor (Ghasemian et al. 2019).

Although CGA inhibited HPU with an IC_50_ approximately twice that of the positive control AHA, its inhibition mechanism warrants further discussion. Lineweaver–Burk analysis and molecular docking revealed that CGA simultaneously obstructed substrate access to urease and stabilized the mobile flap. This stabilization may maintain the flap in a partially closed intermediate conformation, which is incompatible with productive substrate binding and catalysis (Mazzei et al. 2020). The inhibitory potency of CGA is likely attributed to its structural scaffold, specifically the catechol and polyhydroxyl moieties. These multiple hydrogen‐bond donors facilitate simultaneous interactions with both the active site and the flap region (Lu et al. 2022).

Here, several complementary assays were applied to explore the binding affinity of the CGA–HPU interaction, alongside the characterization of the quenching mechanism, thermodynamic driving forces, and real‐time binding kinetics. Fluorescence quenching experiments confirmed that CGA interacted with HPU to form a stable complex through a static quenching mechanism (*K*_q_ significantly exceeded 1 × 10^10^ M^−1^ s^−1^ (the diffusion‐controlled limit)). The affinity may be related to the chemical structure of CGA, specifically its catechol and polyhydroxyl moieties (Haq et al. 2024). The binding constant (*K* = 2.19 ± 0.14 × 10^7^ M^−1^) was 2–3 orders of magnitude higher than the values (*K*_a_ and *K*_A_) yielded by ITC and SPR. These discrepancies likely stem from methodological differences. While fluorescence quenching localized microenvironmental changes around tyrosine and tryptophan residues (Jha et al. 2023), the binding affinities from ITC and SPR are derived from fitting thermodynamic and kinetic isotherms, respectively (Migliore et al. 2022).

ITC provided direct thermodynamic parameters of CGA–HPU complex formation. Following optimized baseline integration, fitting the raw isotherm with a single‐site binding model yielded a stoichiometry (*N* = 1.09) that closely aligned with the fluorescence‐derived value (*n* = 1.30). The negative values of Δ*G* indicated that the binding process was spontaneous, while the negative Δ*H* indicated that the interaction was predominantly enthalpy‐driven (Zhao et al. 2019; Li et al. 2024). Together with the docking and molecular dynamics results, the ITC data support that hydrogen bonding and van der Waals interactions formed by the hydroxyl‐rich structure of CGA contribute to stable complex formation and urease inhibition (Guan et al. 2024). Tang et al. found that protein conformational remodeling can enhance polyphenol loading by exposing accessible binding sites, with hydrophobic and van der Waals interactions serving as major driving forces (Tang et al. 2026).

SPR was employed to elucidate the binding kinetics between CGA and HPU. The value of *K*_D_ was within the same order of magnitude as the ITC results, indicating a moderate binding affinity. The association rate constant *K*_on_ was found to be in the 10^2^ M^−1^ s^−1^ range, situated at the lower bound of the typical spectrum (10^2^–10^8^ M^−1^ s^−1^) for small molecule‐protein interactions (Schoop and Dey 2015). These kinetic data revealed that the CGA–HPU interaction proceeds via a non‐diffusion‐limited mechanism, where the complex must overcome a major energy barrier associated with the open‐to‐closed conformational transition of the flap to form a stable complex (Copeland et al. 2006).

Molecular dynamics simulations were performed to explore the inhibitory mechanism at the molecular level. The CGA–HPU complex reached a stable equilibrium as evidenced by converged RMSD values during the 50 ns simulation. Compared to the apo‐enzyme, the complex exhibited significantly lower RMSD and fewer fluctuations, indicating that CGA binding enhances the conformational stability of HPU (Prabhakaran et al. 2023). Low RMSF values (< 0.25 nm) further confirmed the high structural stability of the complex. Key residues such as Asp223 and Met366 exhibited minimal fluctuations, consistent with their role in forming stable hydrogen bonds and van der Waals interactions within the HPU binding pocket (Parvez et al. 2022). This stability is also reflected in the Rg profiles. The marginal shift in Rg (from 1.78 to 1.84 nm) suggested a subtle conformational adjustment to accommodate the ligand while maintaining overall compactness. MM–PBSA analysis yielded a total binding free energy (Δ*G*) of −16.63 ± 3.57 kcal/mol, further supporting the spontaneous nature of the binding process (Das et al. 2023). However, the 50 ns molecular dynamics simulation is relatively brief and may not fully capture large‐scale, long‐term conformational transitions of HPU. Furthermore, our results are based on in vitro experiments only; further in vivo experiments will be necessary to confirm the HPU inhibitory effects of CGA.

## 5 Conclusion

This study systematically elucidated the inhibitory mechanism of CGA against HPU through integrated enzymatic, biophysical, and computational analyses. CGA exhibited mixed‐type inhibition and formed a stable complex with HPU, a process driven primarily by favorable enthalpic contributions. Structural analyses further revealed that CGA interacted with key residues within the active site and the adjacent binding site, contributing to the stability of the complex.

## Author Contributions

**Hongguang Cao:** validation. **Yanni Li:** conceptualization, writing – review and editing, methodology. **Luzhe Wu:** conceptualization, methodology, writing – original draft. **Ning Xu:** data curation. **Chen Yu:** conceptualization, funding acquisition, writing – review and editing. **Shengyao Hou:** methodology. **Chenyu Wang:** methodology, formal analysis.

## Funding

This project was supported by the National Natural Science Foundation of China [No. 81903697]; 2024 Science and Technology Innovation Project of the “Special Program for Traditional Chinese Medicine”, Binzhou Medical University (2024ZYYZX18).

## Disclosure

The authors have nothing to report.

## Supporting information


**Figure S1:** Electrophoretogram of SDS‐PAGE of HPU. M, Marker, lane 1, Flow though, lane 2–5 purified samples (20 mM PB, 150 mM NaCl, pH 7.0 elution sample), lane 6–8 purified samples (20 mM PB, 300 mM NaCl, pH 7.0 elution sample).
**Figure S2:** Inhibition effect of chlorogenic acid on HPU activity.
**Table S1:** Effect of different concentrations of chlorogenic acid on the kinetic parameters of HPU.
**Table S2:** Binding free energy decomposition of the CGA–HPU complex.

## Data Availability

The data are contained within the article or Supporting Information S1.
