# Supplementary material for: In Silico and In Vitro Elucidation of the Inhibitory Mechanism of Chlorogenic Acid Against Helicobacter pylori Urease
Source: Food Sci Nutr. 2026 Jun 28;14(7):e72065. doi: 10.1002/fsn3.72065 (PMC13311192; doi:10.1002/fsn3.72065)
Supplement: Supplementary file 1 — Figure S1: Electrophoretogram of SDS‐PAGE of HPU. M, Marker, lane 1, Flow though, lane 2–5 purified samples (20 mM PB, 150 mM NaCl, pH 7.0 elution sample), lane 6–8 purified samples (20 mM PB, 300 mM NaCl, pH 7.0 elution sample). Figure S2: Inhibition effect of chlorogenic acid on HPU activity. Table S1: Effect of different concentrations of chlorogenic acid on the kinetic parameters of HPU. Table S2: Binding free energy decomposition of the CGA–HPU complex. [file FSN3-14-e72065-s001.docx]

Supplementary Table 1 Effect of different concentrations of chlorogenic acid on the kinetic parameters of HPU

| Concentration (μM) | K_m_ (mM) | V_max_ (mM/min) |
| --- | --- | --- |
| 0 | 0.54±0.01^c^ | 1.04±0.02^a^ |
| 6.25 | 0.57±0.01^bc^ | 0.83±0.03^b^ |
| 12.5 | 0.60±0.02^b^ | 0.76±0.03^bc^ |
| 25 | 0.66±0.03^a^ | 0.68±0.04^c^ |

Values with different letters in the same column represent statistically significant differences (*p* < 0.05)


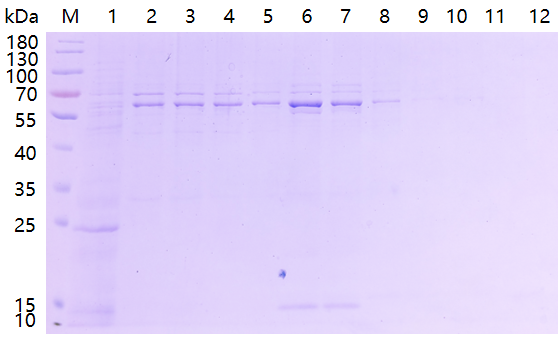


Supplementary Figure 1 Electrophoretogram of SDS-PAGE of HPU. M, Marker, lane 1, Flow though, lane 2-5 purified samples (20 mM PB, 150 mM NaCl, pH 7.0 elution sample), lane 6-8 purified samples (20 mM PB, 300mM NaCl, pH 7.0 elution sample).


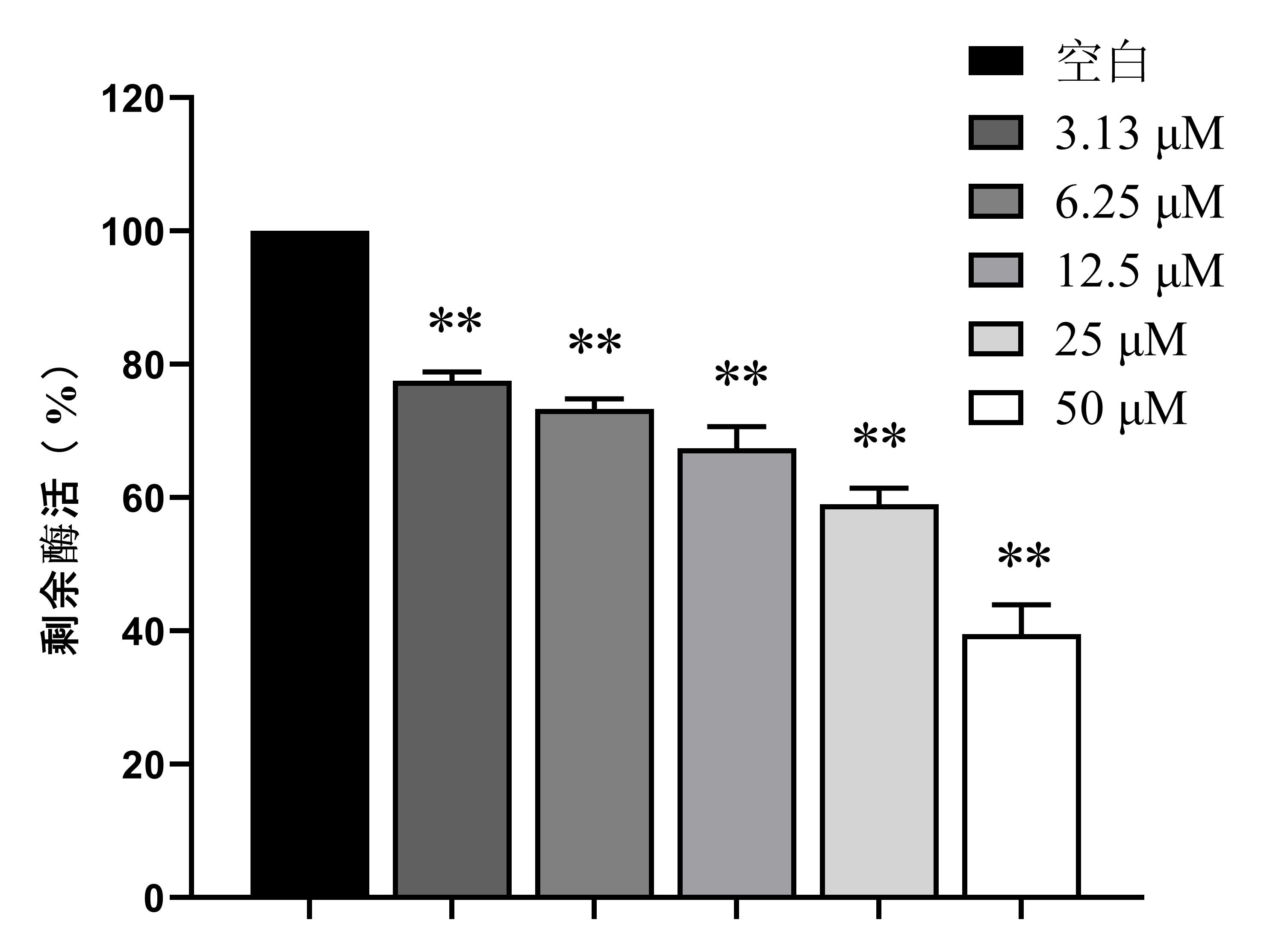


Supplementary Figure 2 Inhibition effect of chlorogenic acid on HPU activity

Supplementary Table 2 Binding free energy decomposition of the CGA**–**HPU complex

| VDWAALS  (kcal/mol) | EEL  (kcal/mol) | EGB  (kcal/mol) | ESURF  (kcal/mol) | GGAS  (kcal/mol) | GSOLV  (kcal/mol) | TOTAL  (kcal/mol) |
| --- | --- | --- | --- | --- | --- | --- |
| −27.78±2.84 | −24.52±5.64 | 39.75±4.43 | −4.08±0.40 | −52.30±5.67 | 35.67±4.52 | −16.63±3.57 |

VDWAALS, van der Waals energy; Eel, Electrostatic energy; EGB, polar solvation energy; ESURF, Non-polar solvation energy; GGAS, total gas phase free energy; GSOLV, total solvation free energy; TOTAL, GSOLV + GGAS.
